# Smart, Naturally-Derived Macromolecules for Controlled Drug Release

**DOI:** 10.3390/molecules26071918

**Published:** 2021-03-29

**Authors:** Izabela Zaborniak, Angelika Macior, Paweł Chmielarz

**Affiliations:** 1 Department of Physical Chemistry, Faculty of Chemistry, Rzeszow University of Technology, Al. Powstańców Warszawy 6, 35-959 Rzeszów, Poland; d410@stud.prz.edu.pl; 2 Doctoral School of Engineering and Technical Sciences at the Rzeszow University of Technology, Al. Powstańców Warszawy 8, 35-959 Rzeszów, Poland; d519@stud.prz.edu.pl

**Keywords:** troxerutin, smart delivery systems, ATRP, polyelectrolytes

## Abstract

A series of troxerutin-based macromolecules with ten poly(acrylic acid) (PAA) or poly(2-dimethylaminoethyl methacrylate) (PDMAEMA) homopolymer side chains were synthesized by a supplemental activator and reducing agent atom transfer radical polymerization (SARA ATRP) approach. The prepared precisely-defined structures with low dispersity (*M*_w_/*M*_n_ < 1.09 for PAA-based, and *M*_w_/*M*_n_ < 1.71 for PDMAEMA-based macromolecules) exhibited pH-responsive behavior depending on the length of the polymer grafts. The properties of the received polyelectrolytes were investigated by dynamic light scattering (DLS) measurement to determine the hydrodynamic diameter and zeta potential upon pH changes. Additionally, PDMAEMA-based polymers showed thermoresponsive properties and exhibited phase transfer at a lower critical solution temperature (LCST). Thanks to polyelectrolyte characteristics, the prepared polymers were investigated as smart materials for controlled release of quercetin. The influence of the length of the polymer grafts for the quercetin release profile was examined by UV–VIS spectroscopy. The results suggest the strong correlation between the length of the polymer chains and the efficiency of active substance release, thus, the adjustment of the composition of the macromolecules characterized by branched architecture can precisely control the properties of smart delivery systems.

## 1. Introduction

Smart polymers are a class of dynamically developing macromolecules with potential use in life science and pharmaceutical fields. They respond to environmental conditions mimicking the behavior of structures and functions of living organisms to adapt to variations in nature. Stimuli-responsive and switchable polymers react through physical or chemical changes, automatically adapting their properties to even small changes in the microenvironment, e.g., temperature, pH, light, redox potential, etc. [1,2]. As a consequence of their sensitivity to external stimuli, such polymeric materials are widely considered as carriers of active substances, e.g., drugs [1,3], active substances [4,5], and genes [6,7], creating intelligent controlled delivery systems with slow, sustained release of substances.

Macromolecules with branched architectures are privileged as smart polymer materials due to their unique morphologies and properties. Complex macromolecules consist of many linear polymers attached to one core with a large number of chain-end functionalities. Owing to these characteristics, polymers with multiple side chains/arms exhibit some remarkable properties unattainable by simple linear polymers. Considering smart materials, macromolecules with branched architectures have strengthened action as a result of many polymer grafts stabilized by one core compared to their linear counterpart, thus, the ability to form unimolecular micelles, and efficiently transport active substances encapsulated within polymer side chains [8,9,10,11].

Star-like polymers and polymer brushes have found numerous applications as different stimuli-responsive polymers delivering drugs [1,2], and recently they were developed as an efficient active substance delivery system examined in vivo in tomatoes [4]. Drug delivery systems used in a live organism with controlled release of an active substance in individual sections of the digestive system need to be sensitive mostly to pH changes. Several polymers are known to change their conformation upon pH changes. Poly(acrylic acid) (PAA) received in a facile way as a result of poly(*tert*-butyl acrylate) (P*t*BA) acidolysis is a typical polymer that can respond to changes in pH. Under pKA (~4.5) carboxylate groups located in a PAA chain are protonated, and the molecules have a compact and collapsed structure, while in alkaline conditions macromolecules are fully stretched due to deprotonation of carboxyl groups, resulting in electrostatic repulsion between –COO¯ moieties [12,13]. Moreover, PAA does not have a significant effect on the proliferation of living cells, therefore, it exhibits high biocompatibility, and has great potential for use as smart drug delivery systems in the human organism [13,14]. Another type of pH-responsive polymer is poly(2-dimethylaminoethyl methacrylate) (PDMAEMA), which contains tertiary amine functional groups. Conversely to PAA chains, in an acidic medium free amino groups located in PDMAEMA are ionized and the structure is extended, while under alkaline conditions many hydrogen bonds are created among amino groups, and the macromolecules adopt compact conformation [15,16]. Moreover, PDMAMEMA exhibits phase separation at the lower critical solution temperature (LCST) in aqueous media, thus, it is a thermoresponisive polymer [17]. Polycations like PDMAEMA are highly promising non-viral gene delivery agents and are able to site-specifically deliver active substances; however, their use in living organisms is limited due to toxicity against healthy cells [18].

There are many works presenting the use of branched polymers for in vitro controlled release of drugs, e.g., doxorubicin [13,19], curcumin [5,20], etc., upon pH changes. They are also considered carriers for active substances in the plant. Smart materials with agrochemical use are increasingly investigated to improve the efficiency and uptake of pesticide, fungicide and micronutrients, and to avoid damage to plants by acidic or alkaline environment, e.g., due to acid rains, or a large amount of plant protection products that are washed off the leaves. The reduction of pollutants related to the high amount of products used to efficiently protect the plant is possible by controlled release of the active substances in the plant when they are needed—when an unfavorable stimulus acts on the plant [21,22].

To precisely determine the properties of smart materials and to thoroughly drive the action, the knowledge about its chemical structure, thus, its well-defined architecture, is mandatory [23]. Polymers with predetermined structures and branched architecture are widely provided by atom transfer radical polymerization (ATRP) techniques, especially with low catalyst concertation avoiding contamination of the final polymer product [24,25,26,27]. As a result of a precisely controlled equilibrium between active and dormant species driven by catalytic complex, the growth of polymer grafts is precisely controlled and the polymers with predetermined structure and properties are able to receive [28,29,30,31]. Branched polymers are usually prepared by ATRP techniques using the *grafting-from* concept, i.e., the modification of a substrate containing ubiquitous hydroxyl groups with bromine is initially conducted, followed by polymerization of monomers from incorporated ATRP initiation sites [32,33,34].

In order to receive biocompatible and biodegradable structures with great potential for use in live organisms by means of ATRP techniques, naturally-derived substrates are increasingly used as macromolecular cores [35]. Troxerutin is a natural bioflavonoid—a trihydroxyethylated derivative of rutin that can be found in tea, coffee, and a variety of fruits and vegetables [36,37]. As a polyphenol structure, it was successfully modified by the ATRP approach to enhance its lipophilicity and, thus, bioavailability, modifying it with hydrophobic poly(*n*-butyl acrylate) (P*n*BA) chains [35]. The presented work shows the facile route for the use of this naturally-derived flavonoid glycoside for preparing smart polymeric materials, namely pH-responsive polymers for controlled release in a live organism, both human or plants. The novel macromolecules with varying length of PAA or PDMAEMA side chains and troxerutin core were synthesized to examine their pH responsiveness as a function of the lengths of polymer grafts. The quercetin (QC) release profile from monomolecular micelles composed of prepared macromolecules was studied.

## 2. Results and Discussion

### 2.1. Synthesis and Characterization of pH-Responsive Troxerutin-Based Star-Shaped Polymers with Different Arm Lengths

**Figure 1:**
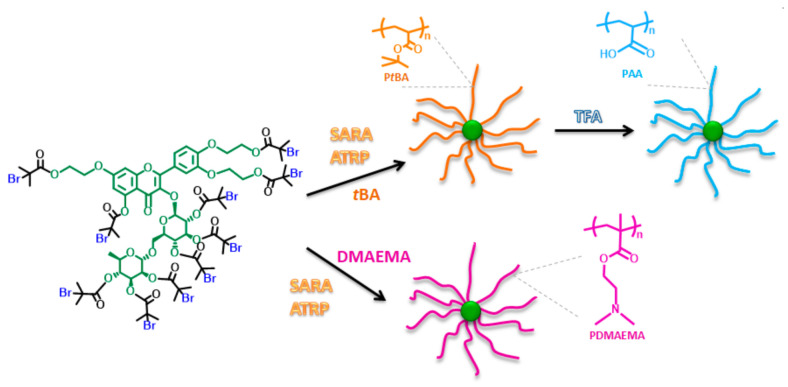
Synthetic route for the preparation of troxerutin-based polyelectrolytes.

Star-like macromolecules with 10 polymer arms and troxerutin core were synthesized by a two-step synthesis route according to the core-first concept (Figure 1).

Hydroxyl groups located in troxerutin substrate were functionalized with α-bromoisobutyryl bromide (BriBBr) to form an ATRP initiator molecule as previously described in detail [35]. The supramolecular bromide initiator was subsequently polymerized with *tert*-butyl acrylate (*t*BA)—an excellent substrate for receiving pH-responsive acrylic acid (AA) moieties in the proposed synthetic route. Acidic hydrolysis with trifluoroacetic acid (TFA) under ambient conditions is widely used for the transformation of *tert*-butyl groups into carboxylic acid [5]. The SARA ATRP approach with the use of copper wire (Cu^0^) with only 165 ppm by weight of catalyst was applied for the synthesis of P*t*BA polymer arms grafted from the troxerutin core. Three types of macromolecules with varying lengths of polymer grafts were received by applying different target degrees of polymerization (DP_target_) in syntheses (Table 1, entry 1–3).

**Figure 2:**
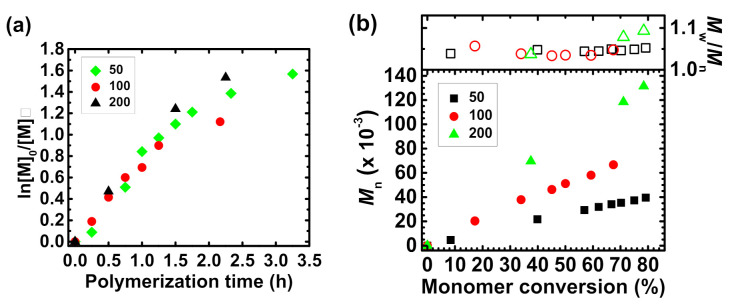
(**a**) First-order kinetics plot of monomer conversion vs. polymerization time of *t*BA from the troxerutin core with different target degrees of polymerization; (**b**) *M*_n_ and *M*_w_/*M*_n_ vs. monomer conversion. Table 1, entry 1–3.

The polymerizations of *t*BA monomer from a naturally-derived core were described by a linear first-order kinetics plot (Figure 2a) and the molecular weight of star-shaped macromolecules steadily grew as monomer conversed (Figure 2b). It indicates a fully controlled syntheses of P*t*BA-based polymers. Moreover, as a result of precisely-controlled processes, polymer products with low dispersity (*M*_w_/*M*_n_ = 1.05–1.09) (Figures S1a, S2a, and S3a) were received. Low molecular weight (LMW) impurities were noticed in the polymerization product (Figures S1b, S2b, and S3b). Their content increased with time, suggesting the transfer to solvent or monomer. However, this phenomenon did not affect the quality of the final macromolecules.

Considering the kinetics aspect of the syntheses, the apparent rate constant of propagation decreases as the concentration of the initiator ([Trox-Br_10_]_0_) increases conversely to the rate of polymerization (*R*_p_) equation [39,40]. This phenomenon is connected with the branched architecture of the received macromolecules. In the preparation of the linear polymers, there is no steric hindrance between growing macromolecules, while during the synthesis of star-shaped polymers the growing polymer side chains/arms located among one core can be entangled and slow down polymer growth, and thus monomer conversion. It is clearly visible on ln[M]_0_/[M] versus the polymerization time plot. At the beginning of the process the rate constants are almost the same, while after ca. 1.5 h the polymerization with higher [Trox-Br_10_]_0_ is slowing down. The received P*t*BA-based macromolecules were hydrolyzed to sensitive to pH changes—PAA moieties. The structures of received polymers with PAA side chains were confirmed by ^1^H NMR and FT-IR analysis (See details in Section S4. Proton nuclear magnetic resonance (^1^H NMR) spectroscopy analysis of P*t*BA-, PAA-, and PDMAEMA-based macromolecules and Section S5. Fourier-transform infrared spectroscopy (FT-IR) analysis of star-shaped polymers).

**Figure 3:**
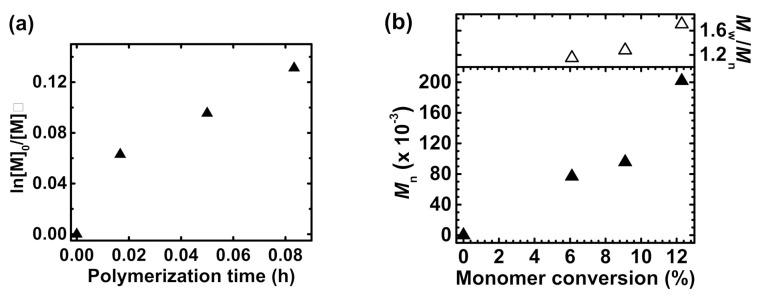
(**a**) First-order kinetic plot of monomer conversion vs. polymerization time of DMAEMA from the troxerutin core; (**b**) *M*_n_ and *M*_w_/*M*_n_ vs. monomer conversion. Table 1, entry 4.

Star-like polymers with troxerutin core and pH responsiveness opposite to PAA-based macromolecules were received by polymerization of DMAEMA from a flavonoid-based initiator. Analogously to PAA-based macromolecules, three different fractions with varying length of polymeric arms were obtained (Table 1, entry 4, Table S6). Deviation from the kinetics plot was observed, suggesting the slowing down of the polymerization as the process proceeds (Figure 3a). While the *M*_n_ vs. monomer conversion curve deviates to higher molecular weight as the monomer is consumed (Figure 3b), it is connected with both characteristics of the ATRP initiator—densely packed initiation sites result in close proximity of polymer arms that terminate due to inter- and intramolecular coupling reaction, and monomer characteristics—DMAEMA can complex a catalyst and disturb the polymerization. Therefore, the received PDMAEMA-based macromolecules are characterized by slightly higher dispersity (*M*_w_/*M*_n_ = 1.15–171) (Figure S4).

The structure of received macromolecules was confirmed by ^1^H NMR and FT-IR analysis (see details in Figure S4. Proton nuclear magnetic resonance (^1^H NMR) spectroscopy analysis of P*t*BA-, PAA-, and PDMAEMA-based macromolecules and Figure S5. Fourier-transform infrared spectroscopy (FT-IR) analysis of star-like polymers).

### 2.2. Stimuli-Responsive Behavior of Troxerutin-Based Polymers

**Figure 4:**
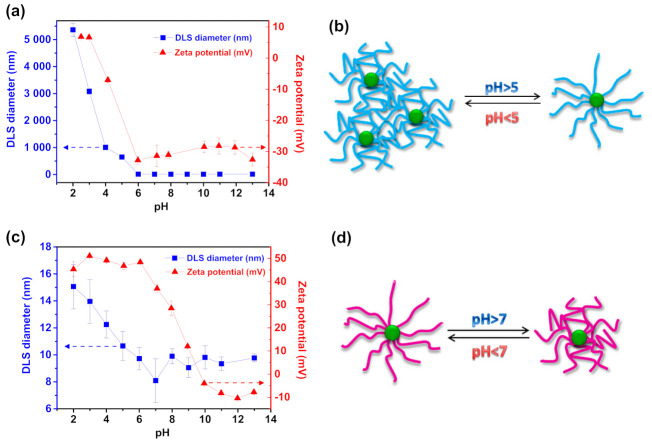
Effect of varying pH on number average sizes and zeta potential of the troxerutin-based macromolecules with (**a**) PAA and (**c**) PDMAEMA side chains; Scheme of pH-responsive behavior of (**b**) PAA and (**d**) PDMAEMA composed macromolecules with troxerutin core. The measurements conducted for final product from the syntheses shown in Table 1, entry 3 and 4, respectively.

Analysis of pH-responsive properties of PAA- and PDMAEMA-based star polymers was conducted by hydrodynamic radius and zeta potential measurements using the DLS method. Poly(acrylic acid) behavior and, thus, solubility in an aqueous solution is strictly connected with the state of ionization of the ubiquitous carboxylic groups present in its structure. Under the pH value ~4.75 (pKa of PAA [41]) carboxylate groups are fully protonated, the polymer has a compact globular conformation and becomes sparingly soluble in water. This follows from the transition of macromolecules characteristics from the hydrophilic to the hydrophobic one. Consequently, PAA-based star-like polymers turn amphiphilic, and the core-shell nanostructures are generated [11,42]. As clearly shown by hydrodynamic diameter measurements of Trox-(PAA-Br)_10_ upon pH changes, below pH ~5 (22 °C) macromolecules were agglomerated, and the generated dispersion was rapidly and macroscopically precipitated (Figure 4b). This resulted in a significant increase in the diameter of nanoparticles (from *d*_number_ ~13 nm, through ~650 nm in pH = 5, to ~5,500 nm in pH = 2, Figure 4a, Table S7). This phenomenon is related to insufficient steric interaction between particles because of non-charged PAA-based chains, thus the van der Waals attractive interactions are not counterbalanced [43,44]. Above pH ~5 (22 °C) the diameter of polymers was *d*_number_ ~11–13 nm, indicating complete molecular dissolution of ionized polymers, and a fully solvated open coil conformation was formed. The results are consistent with ζ-potential measurements. The particles dissolved in alkaline conditions possessed negative surface ζ-potential up to −32 mV. The charge decreased while the pH value decreased reaching ζ-potential equal to 0 mV at the isoelectric point (IEP, pH = 3.56).

A different phenomenon is observed in PDMAEMA-based monomolecular micelles. Although similar to PAA-based macromolecules, namely the higher hydrodynamic diameter value at acidic pH, it is not due to the formation of micelle aggregates. At acidic pH the amine groups present among the PDMAEMA chains are fully protonated, and the polymer chains exhibit high surface charge densities [4,45]. This is proved by ζ-potential measurements results: PDMAEMA macromolecules possessed positive surface ζ-potential up to 51 mV. It increases the electrostatic repulsive forces that exist between the polymer side chains attached to the troxerutin core and the neighboring macromolecules. The hydrophilic polymer side chains self-assemble, avoiding the formation of the micellar aggregations, leading to the fully-expanded polymer chains and, thus, the highest hydrodynamic radius of PDMAEMA-containing monomolecular micelles (Figure 4c,d). As the pH value of an aqueous solution increases, the amine groups become increasingly deprotonated, electrostatic repulsive forces are significantly weaker, and ζ-potential value becomes negative going through 0 mV value in IEP (pH = 9.70). It usually results in micellar growth due to an ability to more efficient packing of macromolecules [46,47,48]. Considering the prepared PDMAEMA-based macromolecules with troxerutin core, the polymer micelles exhibited lower hydrodynamic diameter than in acidic conditions. It suggests the stretching of the DMAEMA-based chains among the troxerutin core as a result of the loss of electrostatic repulsive forces between deprotonated chains, however, it occurs within one densely grafted (10 polymer chains attached to the core) macromolecule, and the aggregates are not formed.

**Figure 5:**
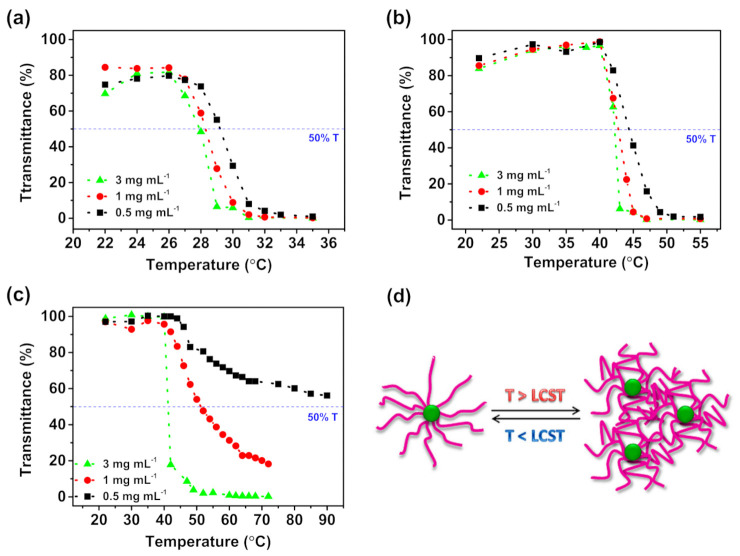
Transmittance at 600 nm for Trox-(PDMAEMA-Br)_10_ with (**a**) 49, (**b**) 73, and (**c**) 98 DMAEMA mers in side chains in aqueous solution with varying concentration; (**d**) Scheme of thermoresponsive properties of PDMAEMA-based macromolecules with a troxerutin core. The measurements conducted for the final products from the syntheses shown in Table 1, entry 4 and Table S5.

Among the pH sensitivity, PDMAEMA also demonstrates phase transition of a polymer solution at a specific concentration—it passes from the soluble state to the collapsed aggregated state when heated above its lower critical solution temperature (LCST, ~47 °C at pH = 7 and ~35 °C at pH = 10), and does not exhibit LCST behavior under acidic conditions [49]. Troxerutin-based macromolecules were examined among LCST considering variations in the length of the polymer grafts and polymer concentration (Figure 5). Thermoresponsive behavior of the prepared macromolecules is strictly influenced by both the length of the PDMAEMA side chains, as the concentration of the macromolecules. Star-like polymers with the shortest side chains exhibited LCST at 27.9, 28.3 and 29.2 °C at concentrations of 3, 1, and 0.5 mg mL^−1^, respectively. The differences in phase transition temperature changed insignificantly (<1 °C) in different troxerutin-based polymers concentration (Figure 5a). Macromolecules with 73 DMAEMA mers in side chains exhibited notably higher LCST values, i.e., 42.2, 42.8 and 44.4 °C at polymers concentration of 3, 1, and 0.5 mg mL^−1^, respectively (Figure 5b). While the molecules with the longest polymer grafts did not show considerable phase transfer at 3 mg mL^−1^ polymer concentration (transmittance of 56% at 90 °C), and demonstrated LCST at 41.2 and 51.5 °C at 3 and 1 mg mL^−1^, respectively (Figure 5c). The results indicate the strong correlation between the phase transition temperature and both the length of the polymer grafts and the concentration of macromolecules, indicating high-temperature stability of the prepared macromolecules with the longest PDMAEMA side chains at 0.5 mg mL^−1^.

The presented results show that macromolecules with branched architecture are more stable than their linear counterparts. Above LCST temperature, PDMAEMA-based molecules are water-insoluble and considering controlled release systems, they can release substances from the micelle structure, while in an alkaline medium PDMAEMA should effectively entrap the encapsulated substance. High LCST value results in avoiding the unexpected release of the substances when the temperature reached high values, thus, the prepared star-shaped polymers could be an effective smart delivery system with sustained release even under harsh temperature conditions, which has a great potential for the use in plants.

### 2.3. In Vitro Drug Release

In view of the pH-responsiveness of the PAA- and PDMAEMA-containing macromolecules, they are capable of a controlled release of an active substance in different pH values. In acidic conditions, PAA polymers are characterized by a compact globular conformation and are able to encapsulate the substances, while PDMAEMA polymer chains are fully protonated and expanded, thus, the encapsulated compound is released under alkaline conditions.

Quercetin, as a model drug with hydrophobic characteristics, was encapsulated by polymer monomolecular micelles. The mechanism of carrier/drug interaction is covered by the hydrophobic interaction possibly contribute to the compatibility between the drug and carrier. QC is a hydrophobic drug with poor solubility in water [50,51]. The prepared polymers are characterized by a unique core-shell architecture, which means the hydrophobic part (troxerutin core) provides a space for the encapsulation of hydrophobic drugs and is surrounded by hydrophilic polymer chains (PAA or PDMAEMA). Polymeric micelles protect the drug (hydrophilic part of micelles), also enabling the delivery of the drug to the desired site at a concentration exceeding the intrinsic solubility, and its sustained release. Considering loading efficiency, PAA-based monomolecular micelles entrapped 85–93% of QC, varying slightly with the length of the macromolecule side chains. While PDMAEMA-based polymers with shorter side chains entrapped the QC more efficiently (EL = 69 and 74% for molecules with the shortest and medium length side chains), the longest side chains entrapped QC two-fold weaker (EL = 30%). As the mechanism of the drug encapsulation is based on the hydrophobic interaction between the drug and the core of the macromolecules, access to the hydrophobic core and steric hindrance caused by the hydrophilic shell has paramount importance. The results indicate that the longest PDMAEMA side chains hinder the QC encapsulation, compared to the other two macromolecules-based micelles.

**Figure 6:**
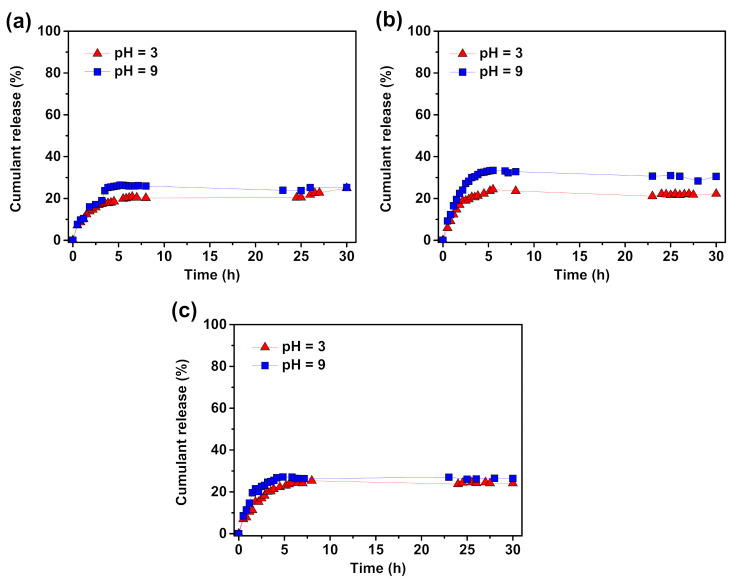
In vitro release profile of quercetin from QC-loaded monomolecular micelles composed of macromolecules with (**a**) 40, (**b**) 67, and (**c**) 157 AA mers in side chains incubated in aqueous solutions at 37 °C. The measurements conducted for the final products from the syntheses shown in Table 1, entry 1–3.

The in vitro release of quercetin was carried out by incubating QC loaded PAA- and PDMAEMA-based monomolecular micelles (Table S9) at 37 °C in buffers with pH values of 3.0 and 9.0, respectively. As illustrated in Figure 6, the release of QC from QC-loaded polymer micelles was pH-dependent. However, the release rate did not increase markedly as the pH changed from 3.0 to 9.0 with increasing lengths of polymer side chains attached to the troxerutin core.

With the increase of the PAA-based side chains of star-shaped polymers, a more visible difference in QC release in various pH was noticed. Under alkaline conditions PAA monomolecular micelles formed by macromolecules with homopolymer side chains composed of 40 (Figure 6a), 67 (Figure 6b), and 157 (Figure 6c) AA mers released 25, 31, and 28% QC after 30 h of incubation in buffers with different pH values, respectively. As the length of the side chains increased, only a few percent more quercetin was released. Under acidic conditions, where the QC should be effectively encapsulated, the efficiency of encapsulation was slightly dependent on the PAA side chains. Monomolecular micelles formed from troxerutin-based polymers with 40 AA subunits in side chains inefficiently entrapped QC, 25% of QC was released. This is the same amount of released active substance as in alkaline conditions. A similar amount of quercetin was released from the medium length and the longest polymer chains, 22 and 24%, respectively. It is merely 8% and 4% less than QC release under alkaline conditions. However, compared to the monomolecular micelles created by polymers with the shortest side chains the differences in the active substance release in different pH were noticed. Therefore, the results show the dependence of the controlled release of QC from PAA-based micelles from the length of polymer grafts.

**Figure 7:**
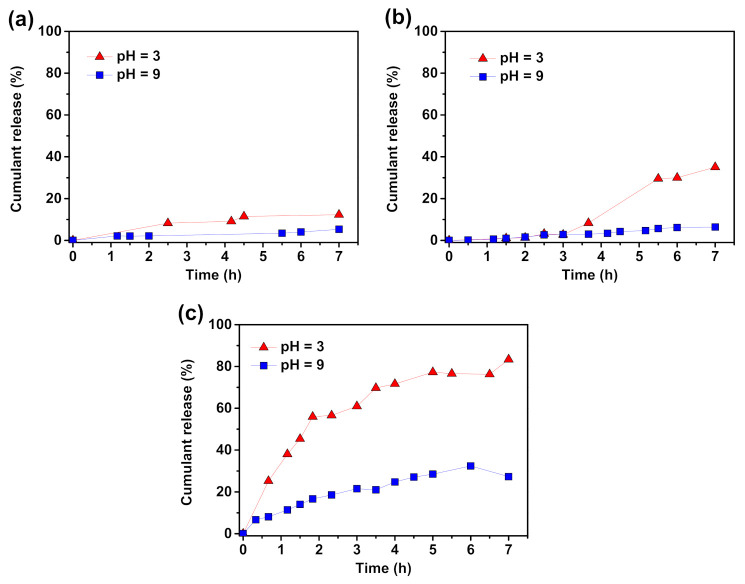
In vitro release profile of quercetin from QC-loaded monomolecular micelles composed of macromolecules with (**a**) 49, (**b**) 73, and (**c**) 98 DMAEMA mers in side chains incubated in aqueous solutions at 37 °C. The measurements conducted for kinetics samples from the synthesis shown in Table 1, entry 4; details in Table S6.

Cumulative release (%) profiles for the QC-loaded PDMAEMA-based monomolecular micelles composed of polymers with troxerutin core with varying length of side chains were investigated during 7 h of incubation in buffers with pH values of 3.0 and 9.0 (37 °C). PDMAEMA-composed micelles exhibited higher efficiency in the controlled release of QC comparing to PAA composed ones (Figure 7).

The release of QC from QC-loaded monomolecular micelles was pH-dependent, and the release rate increased markedly as the pH decreased from 9.0 to 3.0, conversely to PAA-based micelles. Additionally, the results indicate that the cumulative QC release (%) is strictly connected with the length of the polymer grafts, and is significantly higher for the samples with longer PDMAEMA side chains. Under acidic conditions, hydrogen bonds between amino groups are broken, and the PDMAEMA-based molecules exist in a stretched conformation, thus, the release of the active substance is possible. In pH 3.0 the monomolecular micelles formed by star-shaped macromolecules with the shortest side chains (DP_per chain_ = 49) released only 12% of its active substance content, while longer PDMAEMA polymer grafts (DP_per chain_ = 73 and 98) released 35 and 83%, respectively. In alkaline medium micelles effectively entrapped QC due to its compact structure resulted from the presence of hydrogen bonds between tertiary amine groups. About 5, 6, and 27% of the substance was released from the micelles composed from short, medium, and the longest PDMAEMA side chains, respectively. The results showed the great potential of troxerutin-based macromolecules with pH-sensitive grafts in the sustained release of active substances, which can be effectively controlled by selecting the appropriate length of the side chains.

## 3. Materials and Methods

### 3.1. Chemicals

Troxerutin (Trox, *M*_n_ = 742.68, EP Reference Standard), 2-bromoisobutyryl bromide (BriBBr, 98%), *N*-methyl-2-pyrrolidone (NMP, >99%), tetrahydrofuran (THF, >99%), copper(II) bromide (Cu^II^Br_2_, 99.9%), dichloromethane (DCM, >99.9%) and trifluoroacetic acid (TFA, >99%) were purchased from Sigma Aldrich (Saint Louis, MO, USA). *N*,*N*-Dimethylformamide (DMF, 99.9%) was purchased from Acros (Fair Lawn, NJ, USA). Deionized water (ACS Reagent) and sodium hydroxide (NaOH, >98%) were purchased from Honeywell Riedel-de Haen (Seelze, Germany). These reagents were not subjected to further purification. Tris(2-pyridylmethyl)amine (TPMA) and Cu^II^Br_2_/TPMA catalyst complex were prepared as previously reported [35,52]. 2-Dimethylaminoethyl methacrylate (DMAEMA, >99%, Sigma-Aldrich) and *tert*-butyl acrylate (*t*BA, >99%, Sigma-Aldrich) were passed through a column filled with basic alumina before use in order to remove inhibitor [53]. Cu^0^ wire was purchased from Alfa Aesar (99.9%, Tewksbury, MA, USA).

### 3.2. Analysis

Proton nuclear magnetic resonance (^1^H NMR) spectra were carried out in CDCl_3_ using Bruker Avance 500 MHz spectrometer (Bruker, Karlsruhe, Germany) in 25 °C. Number-average molecular weight (*M*_n_) and molecular weight distribution (*M*_w_/*M*_n_) were measured by size exclusion chromatography GPC using a Shimadzu (Kyoto, Japan) modular system equipped with a CBM-40 system controller, SIL-20AHT automatic injector, two Repro-Gel GPC 5 μm columns (10,000 and 100,000 Å) and RID-20A differential refractive-index detector. The temperature of the columns was maintained at 30 °C using a CTO-20A oven. The eluent was *N,N*-dimethylformamide (HPLC grade, with 0.05 M LiCl) and the flow rate was kept at 1 mL min^−1^ using an LC-40 pump. A molecular weight calibration curve was produced using commercial narrow molecular weight distribution polystyrene standards (PSS Polymer Standards Service, Mainz, Germany). Number mean diameter (*d*_number_) of polymer sample (5 mg/mL in THF or water) and zeta potential was measured by dynamic light scattering (DLS, Zetasizer Nano ZS, Malvern Panalytical, Worcestershire, UK) at 22 °C. The UV–VIS spectra were obtained on a Hewlett-Packard (Waldbronn, Germany) Model HP-8453 diode array rapid scan spectrophotometer using a quartz cell with optical length of 1 cm. FT-IR analyses were conducted with the spectrophotometer Nicolet 6700 FT-IR (Thermo Scientific, Waltham, MA, USA), within 500–4000 cm^−1^, with the use of attenuated total reflectance (ATR) technique.

### 3.3. Synthesis of Troxerutin-Based Macroinitiator (Trox-Br_10_)

Troxerutin-based ATRP macroinitiator was prepared according to the previous published procedure [35] as follows: troxerutin (1 g, 1.35 mmol) was dissolved in NMP (20 mL) under Ar atmosphere in 50 mL round-bottom flask equipped with 25 mL cylindrical separatory funnel filled with a solution of BriBBr (4.99 mL, 40.4 mmol, four-fold molar excess to each troxerutin hydroxyl group) in NMP (12.0 mL). A solution was added dropwise over a period of 1 hour at 0 °C, and the reaction mixture was stirred for seven days at room temperature. The reaction mixture was dissolved in 40 mL dichloromethane and washed with 60 mL water (1×) and 60 mL sodium bicarbonate (6×). The product was dialyzed against water six times using a molecular cut-off membrane (MWCO 1000) for 14 days. Then water was removed under pressure and the resulting brown powder was dried over 24 h under vacuum (53.9 mg, yield 35.9%).

### 3.4. General Procedure for SARA ATRP of tBA from Trox-Br_10_

The mixture of *t*BA (3.00 mL, 20.5 mmol), Cu^II^Br_2_/TPMA stock solution (123 µL of 0.05 M in DMF), Trox-Br_10_ (91.5 mg, 4.1 mmol or 45.7 mg, 2.0 mmol, or 22.9 mg, 1.0 mmol) and DMF (5.88 mL) was added to a 10 mL Schlenck flask equipped with a magnetic stirrer bar. Next, Cu^0^ wire (dimension = 10 cm length; thickness 1 mm) previously activated with HCl and washed with THF was placed in a rubber septum that was placed in a Schlenck flask containing reaction mixture. The mixture was degassed for 15 min under argon atmosphere. The flask was placed in an oil bath heated to 50 °C. The polymerization reaction was initiated after adding Cu^0^ wire to the reaction mixture. Samples were withdrawn periodically to follow monomer conversion using ^1^H NMR analysis, and to check *M*_n_ and *M*_w_/*M*_n_ of the polymers by GPC analysis. Before GPC analysis the polymer samples were dissolved in DMF + 0.05 M LiCl + toluene as external standard mobile phase, and passed through a neutral alumina column with 0.22 µm syringe filter in order to remove the catalyst. The polymerization was stopped by opening the flask and exposing the catalyst to air after t = 3.25 h (Table 1, entry 1), t = 2.17 h (Table 1, entry 2), and t = 2.25 h (Table 1, entry 3). The final polymer product was purified by precipitation against MeOH and dried under vacuum, and analyzed by ^1^H NMR.

### 3.5. Transformation of P*t*BA to PAA Side Chains

The P*t*BA moieties of troxerutin-based macromolecules were hydrolyzed to PAA blocks as previously reported [5]. The final sample was characterized by ^1^H NMR and FT-IR analysis.

### 3.6. General Procedure for SARA ATRP of DMAEMA from Trox-Br_10_

The mixture of DMAEMA (6.00 mL, 17.8 mmol), Cu^II^Br_2_/TPMA stock solution (0,499 µL of 0.05 M in DMF), Trox-Br_10_ (10 mg, 2.0 mmol), and DMF (13.50 mL) was added to 20 mL Schlenck flask equipped with a magnetic stirrer bar. Then, Cu^0^ wire (*l* = 20 cm, *d* = 1 mm) previously activated with HCl and washed with THF was placed in a rubber septum that was placed in a Schlenck flask containing the reaction mixture. The mixture was degassed for 25 min under an argon atmosphere. The flask was placed in an oil bath heated to 50 °C. Cu^0^ wire was added to the reaction flask to start the reaction polymerization. Samples were withdrawn periodically to follow the monomer conversion, using ^1^H NMR. The molecular weight and molecular weight distribution of the polymers were measured by size exclusion chromatography (SEC). To receive three kinetics samples, three syntheses were conducted in the same reaction conditions stopping the polymerizations after 1, 3, and 5 min. The polymerization was stopped by opening the flask and exposing the catalyst to air. The polymer products were purified by dialysis against water and dried under vacuum, and analyzed by ^1^H NMR.

### 3.7. Determination of pH-sensitivity of PAA- and PDMAEMA-Based Polymers

The behavior of the troxerutin-based macromolecules with PAA side chains against pH changes was investigated by potentiometric titration, determining the hydrodynamic radius of polymers and zeta potential by DLS measurements of the polymer solutions in different pH values at 22 °C. Potentiometric titration was performed using a digital pH meter (CPC-551, ELMETRON, Zabrze, Poland) equipped with a combined glass/reference electrode (ERH-13-6, HYDROMET, Gliwice, Poland). The PAA-based polymer was dissolved in 0.1 M NaOH (3 mg/mL solution). The value of pH was adjusted by titration with aqueous 0.1 M HCl under intensive stirring, at a constant temperature (22 °C). While PDMAEMA-based polymer was dissolved in 0.1 M HCl and the value of pH was adjusted by titration with aqueous 0.1 M NaOH under intensive stirring, at a constant temperature (22 °C). pH readings were registered after each portion of titrant added to the polymer solution when the equilibrium state was established and the polymer sample was analyzed by DLS measurement.

### 3.8. Determination of Thermoresponsive Behavior of PDMAEMA-Based Polymers

Thermoresponsive behavior of the troxerutin-based macromolecules with DMAEMA side chains was investigated by measurements of transmittances (UV–VIS measurements) of the polymer aqueous solutions (carbonate buffer pH = 9) at various temperatures and different polymer concentration. Transmittances were measured at a wavelength of 600 nm, and the lower critical solution temperature (LCST) value of the lignin-based polymer solution was defined as the temperature producing a 50% decrease in transmittance.

### 3.9. Loading of Quercetin into the PAA- and PDMAEMA-Based Polymer and Release Behavior upon pH Changes

Quercetin loaded micelles were prepared by the dialysis method namely, 20 mg of troxerutin-based polymer and 8 mg of QC was dissolved in 5 mL DMF separately, stirred for half an hour and then mixed and stirred for half an hour. The mixed solution was added dropwise into 10 mL water and stirred for 1.5 h. The prepared micelle solution was placed in a dialysis membrane (Spectra/Por dialysis membrane, MWCO 1000) and dialyzed against deionized water for some cycles over seven days. The received QC loaded micelles solution was adjusted to 60 mL by DMF or water, and the total mass of QC loaded in PAA-based micelles was estimated on the basis of the calibration curve of the absorption intensity at λ = 374 nm as a function of QC concentration in DMF. The loading efficiency (EL) was calculated as follows:

(1) $$\mathrm{EL}\;(\mathrm{wt.}\%)=\frac{w_{p}}{w_{t}}\times 100\%$$

where w_p_ and w_t_ are the mas of QC in micelles and total QC used in the preparation of micelles, respectively (Table S9).

To investigate the release of an active substance loaded in PAA- or PDMAEMA-based micelles 20 mL of QC loaded micelle solution was placed into a dialysis membrane (Spectra/Por dialysis membrane, MWCO 1000) and immersed into 300 mL of buffer solution (citrate-phosphate buffer pH = 3 and carbonate buffer pH = 9). The solutions were incubated at 37 °C. The samples were withdrawn periodically 1 mL and replaced by 1 mL of water. The concentration of released QC was investigated on the basis of UV–VIS spectra based on calibration curves of the absorption intensity at λ = 366 (pH = 3) and λ = 320 (pH = 9) as a function of QC concentration in an appropriate buffer.

## 4. Conclusions

Precisely-defined polyelectrolytes with troxerutin core and ten PAA or PDMAEMA arms were synthesized by SARA ATRP approach using metallic copper as a reducing agent. PAA-based polymers were formed by acidolysis of P*t*BA moieties, and the structure was successfully confirmed by ^1^H NMR and FT-IR analysis. The prepared macromolecules were characterized by narrow molecular weight distribution: *M*_w_/*M*_n_ = 1.05–1.09 and *M*_w_/*M*_n_ = 1.15–1.71 for P*t*BA and PDMAEMA-based polymers, respectively. Dynamic light scattering measurements demonstrate the pH-responsive behavior of both PAA and PDMAEMA composed star-shaped polymers. In acidic conditions, Trox-(PAA-Br)_10_ molecules were agglomerated, and the generated dispersion was rapidly and macroscopically precipitated, resulting in a significant increase in the diameter of nanoparticles (from *d*_number_~13 nm to *d*_number_ ~5500 nm), while in alkaline medium the polymers were fully soluble in aqueous solution. PDMAEMA-based monomolecular micelles exhibited fully expanded polymer chains in acidic pH, thus, the highest hydrodynamic radius. With the increase of pH value, the polymers were stretched and the hydrodynamic radius was decreased, without forming the aggregates.

The in vitro release of quercetin was carried out by incubating QC loaded PAA- and PDMAEMA-based monomolecular micelles at 37 °C in buffers with pH values of 3.0 and 9.0, respectively. The release of QC from QC-loaded polymer micelles was pH-dependent and was more efficient with longer polymer grafts. Considering PAA-based monomolecular micelles, the polymer grafts length has an inconsiderable influence on release efficiency. However, polymer micelles composed of molecules with the longest side chains more efficiently entrapped QC inside. While cumulative QC release (%) from PDMAEMA-based monomolecular micelles was more efficient and was strictly connected with the length of the polymer grafts, the micelles with the longest polymer grafts efficiently released 83% of QC in acidic conditions, and entrapped the substance in alkaline solutions, releasing only 35% of QC. Additionally, the LCST value of PDMAEMA star-shaped polymers was higher than in their linear counterparts presented in the literature, thus, the received polymers are able to efficiently entrapped the substances even at harsh temperature conditions, and have excellent potential for use as a smart delivery system in plants. The results showed the great potential of troxerutin-based macromolecules with pH-sensitive grafts in sustained release of active substances, which can be effectively controlled by selecting the appropriate length of the side chains in both human organism and plants. The star-like polymers with PAA side chains due to a fully biocompatible both troxerutin core and acrylic acid-based grafts have a great potential for the use in human organism for smart site-specific delivery of drug and active substances. While PDMAEMA exhibits toxicity on healthy living cells, thus it can be used as smart materials with agrochemical use to efficiently release pesticide, fungicide, and micronutrients, and avoid the damage of plant by acid rains or a large amount of plant protection products that are washed off the leaves.

## Figures and Tables

**Table 1:** Synthesis of star-shaped macromolecules composed of troxerutin core and P*t*BA or PDMAEMA side chains via the SARA ATRP technique.

Entry	Monomer	DP_target_	Conv ^1^ (%)	*k*_p_^app 2^ (h^−1^)	DP_n_,_theo_^1^ (per chain)	*M*_n,theo_^3^ (×10^−3^)	*M*_n,app_^4^ (×10^−3^)	*M*_w_/*M*_n_^4^	*d*_number_^5^ (nm)	*d*_number_^6^ (nm)
1	*t*BA	50	79	0.594	40	53.0	39.5	1.05	7.7 ± 0.7	3.4 ± 0.3
2	*t*BA	100	67	0.609	67	88.6	66.7	1.05	10.6 ± 0.7	4.3 ± 0.2
3	*t*BA	200	78	0.734	157	203.3	131.4	1.09	17.1 ± 1.8	6.6 ± 0.7
4	DMAEMA	800	12	1.723	98	156.7	201.9	1.71	-	7.2 ± 0.9 ^7^

^1^Monomer conversion (Conv) and theoretical degree of polymerization (DP_n_,_theo_) calculated according to ^1^H NMR analysis, DP_n_,_theo_ = (Conv × [M]_0_)/[Trox-Br_10_]_0_, where M denote monomer [38]; ^2^ Apparent rate constant of propagation, calculated as a slope of the curve ln[M]_0_/[M] = *f(t*) illustrated in the Figure 2a and Figure 3a [38]; ^3^ *M*_n,theo_ = [M]_0_/[Trox-Br_10_]_0_ × Conv × *M*_monomer_ + *M*_Trox-Br10_; ^4^ Apparent *M*_n,app_ and *M*_w_/*M*_n_ were determined by DMF GPC; ^5^ Number mean diameter (*d*_number_) of P*t*BA-based polymers measured by DLS (in THF) after purification (Figure S9a,c,e); ^6^ Number mean diameter (*d*_number_) of PAA-based polymers measured by DLS (in deionized water) after purification (Figure S9b,d,f); ^7^ Number mean diameter (*d*_number_) of PDMAEMA-based polymers measured by DLS (in deionized water) after purification (Figure S10).

## Data Availability

The data presented in this study are available on request from the corresponding author.

## Supplementary Materials

The following are available online, Figure S1. GPC traces of Trox-(P*t*BA-Br)_10_ polymer samples withdrawn periodically during polymerization with DP_target_ = 50: (a) High-molecular weight (HMW) fraction of Trox-(P*t*BA-Br)_10_ and (b) with an additional low-molecular weight (LMW) fraction formed during the polymerization, Figure S2. GPC traces of Trox-(P*t*BA-Br)_10_ polymer samples withdrawn periodically during polymerization with DP_target_ = 100: (a) High-molecular weight (HMW) fraction of Trox-(P*t*BA-Br)_10_ and (b) with an additional low-molecular weight (LMW) fraction formed during the polymerization, Figure S3. GPC traces of Trox-(P*t*BA-Br)_10_ polymer samples withdrawn periodically during polymerization with DP_target_ = 200: (a) high-molecular weight (HMW) fraction of Trox-(P*t*BA-Br)_10_ and (b) with an additional low-molecular weight (LMW) fraction formed during the polymerization, Figure S4. GPC traces of Trox-(PDMAEMA-Br)_10_ kinetics polymer samples, Figure S5. ^1^H NMR spectrum of macromolecules with a troxerutin core and P*t*BA side chains (Table 1, entry 3; *M*_n_ = 203,300, *M*_w_/*M*_n_ = 1.09) after purification (in CDCl_3_), Figure S6. ^1^H NMR spectrum of macromolecules with troxerutin core and PAA side chains received as a result of acidolysis of *t*BA moieties (Table 1, entry 3; *M*_n_ = 203,300, *M*_w_/*M*_n_ = 1.09) after purification (in CDCl_3_), Figure S7. ^1^H NMR spectrum of macromolecules with troxerutin core and PDMAEMA side chains (Table 1, entry 3; *M*_n_ = 203,300, *M*_w_/*M*_n_ = 1.09) after purification (in CDCl_3_), Figure S8. FT-IR spectra of troxerutin modified with bromine (ATRP macroinitiator), P*t*BA (Table 1, entry 3), PAA (hydrolyzed, Table 1, entry 3) and PDMAEMA (Table 1, entry 4), Figure S9. DLS analysis of P*t*BA (a), (c), (e) and corresponding PAA (b), (d), (f) received in the polymerization with the DP_target_ = 50 (Table 1, entry 1), 100 (Table 1, entry 2) and 200 (Table 1, entry 3), respectively, Figure S10. DLS analysis of PDMAEMA-based macromolecules with side chains composed of (a) 49 (Table S6, entry 1), (b) 73 (Table S6, entry 2), and (c) 98 (Table 1, entry 4, Table S6, entry 3) DMAEMA subunits, Table S1. Calculation of Cu^I^/Cu^II^ ratio for the preparation of troxerutin-based macromolecules, Table S2. Calculation of theoretical dead chain fraction (DCF_theo_) for polymerization of (meth)acrylates via SARA ATRP approach, Table S3. Low-molecular weight (LMW) fraction in the samples withdrawn periodically during polymerization of *t*BA from Trox-Br_10_ with DP_target_ = 50, Table S4. Low-molecular weight (LMW) fraction in the samples withdrawn periodically during polymerization of *t*BA from Trox-Br_10_ with DP_target_ = 100, Table S5. Low-molecular weight (LMW) fraction in the samples withdrawn periodically during polymerization of *t*BA from Trox-Br_10_ with DP_target_ = 100, Table S6. Kinetics polymer samples of DMAEMA polymerization from Trox-Br_10_, Table S7. Number of the mean diameter of PAA-based macromolecules at varying pH, Table S8. Number of the mean diameter of PDMAEMA-based macromolecules at varying pH. References [5,28,35,45,53] are cited in the supplementary materials.

## Sample Availability

Samples of the compounds presented in the paper are available on request from the corresponding author.
