# Supplementary material for: Smart, Naturally-Derived Macromolecules for Controlled Drug Release
Source: Molecules. 2021 Mar 29;26(7):1918. doi: 10.3390/molecules26071918 (PMC8037046; doi:10.3390/molecules26071918)
Supplement: Supplementary file 1 [file molecules-26-01918-s001.pdf]

Supplementary Material

Smart, Naturally-Derived Macromolecules for Controlled Drug Release

Izabela Zaborniak [1], Angelika Macior [2] and Paweł Chmielarz [1,*]

[1] Department of Physical Chemistry, Faculty of Chemistry, Rzeszow University of Technology, Al. Powstańców Warszawy 6, 35-959 Rzeszów, Poland; d410@stud.prz.edu.pl
[2] Doctoral School of Engineering and Technical Sciences at the Rzeszow University of Technology, Al. Powstańców Warszawy 8, 35-959 Rzeszów, Poland; d519@stud.prz.edu.pl
* Correspondence: p_chmiel@prz.edu.pl; Tel.: +48-17-865-1809

Content

S1. Estimation of the Cu^{I}/Cu^{II} ratio during the polymerization processes 3

S2. Calculation of theoretical Dead Chain Fraction (DCF_{theo}) for polymerization of (meth)acrylates *via* SARA ATRP technique 3

S3. GPC analysis of troxerutin-based macromolecules 4

S4. Proton nuclear magnetic resonance (^{1}H NMR) spectroscopy analysis of P*t*BA-, PAA- and PDMAEMA-based macromolecules 6

S5. Fourier-transform infrared spectroscopy (FT-IR) analysis of star-like polymers 8

S6. Hydrodynamic diameter measurements of troxerutin-based polymers by dynamic light scattering (DLS) analysis 10

S7. Stimuli-responsive behavior of troxerutin-based polymers 11

S8. Preparation of PAA- and PDMAEMA-based micelles 12

References 13

S1. Estimation of the Cu^I/Cu^{II} ratio during the polymerization processes

Table S1. Calculation of Cu^I/Cu^{II} ratio for the preparation of troxerutin-based macromolecules.

Entry (according to Table 1)	k_p^{app} (h^{-1})¹	$[P_n^•]$ (M ×10¹⁰)¹	K_{ATRP} (×10⁶)²	$[P_n–Br]$ (mM)	$[Cu^I]/[Cu^{II}]$³	$[Cu^IL^+]$ (%)	$[Br–Cu^{II}L^+]$ (%)
1	0.594	57.69	1.70	4.10	0.83	45.3	54.7
2	0.609	59.15	1.70	2.05	1.70	63.0	37.0
3	0.734	71.29	1.70	1.03	4.09	80.3	19.7
4	1.723	4,038.91	1.70	6.74	35.24	97.2	2.8

¹The radical concentration $[P_n^•]$ was calculated according to the equation defined as $[P_n^•] = \left(\frac{d\ln[M]}{dt}\right)\left(k_p\right)^{-1}$, where $\frac{d\ln[M]}{dt}$ values were calculated from the first order kinetics plots (2a and 3a) [1], entry 1–3: $k_p = 2.86{\cdot}10^4\ M^{-1}s^{-1}$ for *t*BA polymerization [2], entry 4: $k_p = 1.19{\cdot}10^3\ M^{-1}s^{-1}$ for DMAEMA polymerization [1]; ²$K_{ATRP} = 1.7 \cdot 10^{-6}$ was determined theoretically for the $Cu^I/TPMA^+$ catalyst in *N*,*N*-dimethylformamide (50% v/v) with methyl 2-bromopropionate as initiator at 25 °C [1]; ³The Cu^I/Cu^{II} ratio was calculated according to the equation defined as $\frac{[Cu^ITPMA^+]}{[Br-Cu^{II}TPMA^+]} = \frac{[P_n^•]}{[P_n-Br]K_{ATRP}}$ [2].

S2. Calculation of theoretical Dead Chain Fraction (DCF_{theo}) for polymerization of (meth)acrylates *via* SARA ATRP technique

Table S2. Calculation of theoretical Dead Chain Fraction (DCF_{theo}) for polymerization of (meth)acrylates *via* SARA ATRP approach.

Entry (according to Table 1)	$[P_n^•]$ (M ×10¹⁰)¹	[D] (M ×10⁶)²	$[P_n–Br]$ (mM)	DCF_{theo} (%)³
1	57.69	38.94	4.10	0.95
2	59.15	27.29	2.05	1.33
3	71.29	41.17	1.03	4.01
4	4038.91	1223.46	6.74	18.15

¹The radical concentration [P ·] was calculated according to the equation defined as $[P_n^•] = \left(\frac{d\ln[M]}{dt}\right)\left(k_p\right)^{-1}$, where $\frac{d\ln[M]}{dt}$ values were calculated from the first order kinetics plots (Figure 2a and 3a) [3], entry 1–3: $k_p = 2.86{\cdot}10^4\ M^{-1}s^{-1}$ for *t*BA polymerization [2], entry 4: $k_p = 1.19{\cdot}10^3\ M^{-1}s^{-1}$ for DMAEMA polymerization [1]; ²The concentration of terminated chains [D] was calculated according to the equation defined as $[D] = k_t[P\,\cdot]^2 t$ where *t* (denote reaction time) = 11,700 s (entry 1), *t* = 7,801 s (entry 2), *t* = 8,100 s (entry 3) and *t* = 300 s (entry 4), entry 1–3: $k_t = 1.0{\cdot}10^8\ M^{-1}s^{-1}$ [2], entry 4: $k_t = 2.5{\cdot}10^7\ M^{-1}s^{-1}$ [1]; ³$DCF = \left(\frac{[D]}{[P-X]_0}\right) \cdot 100\%$ [2].

S3. GPC analysis of troxerutin-based macromolecules

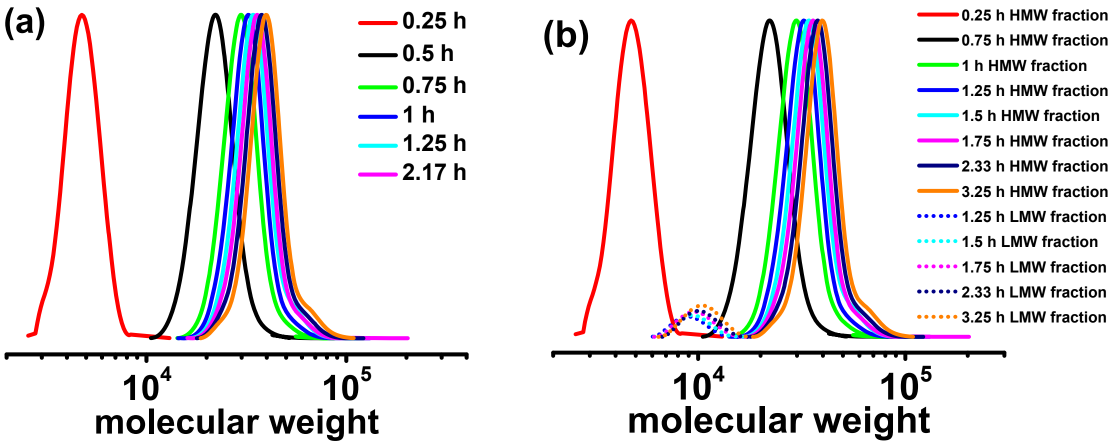

Figure S1. GPC traces of Trox-(P*t*BA-Br)$_{10}$ polymer samples withdrawn periodically during polymerization with DP_{target} = 50: (**a**) High-molecular weight (HMW) fraction of Trox-(P*t*BA-Br)$_{10}$ and (**b**) with an additional low-molecular weight (LMW) fraction formed during the polymerization.

Table S3. Low-molecular weight (LMW) fraction in the samples withdrawn periodically during polymerization of *t*BA from Trox-Br$_{10}$ with DP_{target} = 50.

Sample	% content	$M_{n,app}$[1] (x10^{-3})	M_w/M_n[1]
1	not formed	-	-
2	not formed	-	-
3	not formed	-	-
4	4.01	9.0	1.026
5	4.28	9.4	1.027
6	5.29	9.7	1.030
7	5.34	10.1	1.029
8	6.80	10.4	1.033

[1]Apparent $M_{n,app}$ and M_w/M_n were determined by DMF GPC.

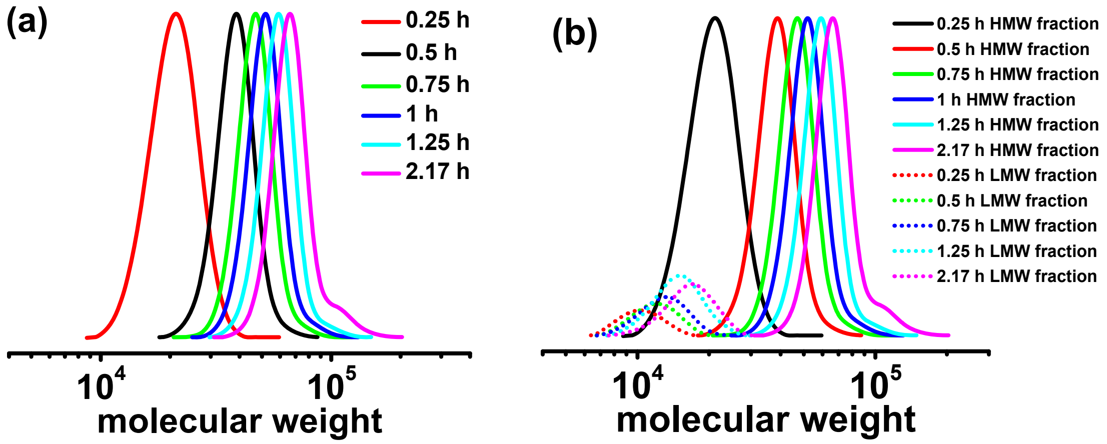

Figure S2. GPC traces of Trox-(P*t*BA-Br)$_{10}$ polymer samples withdrawn periodically during polymerization with DP_{target} = 100: (**a**) High-molecular weight (HMW) fraction of Trox-(P*t*BA-Br)$_{10}$ and (**b**) with an additional low-molecular weight (LMW) fraction formed during the polymerization.

Table S4. Low-molecular weight (LMW) fraction in the samples withdrawn periodically during polymerization of *t*BA from Trox-Br_{10} with DP_{target} = 100.

Sample	% content	$M_{n,app}$[1] (x10^{-3})	M_w/M_n[1]
1	not formed	-	-
2	6.08	10.3	1.038
3	8.24	11.7	1.042
4	10.14	12.7	1.048
5	15.67	14.6	1.054
6	13.17	16.3	1.053

[1]Apparent $M_{n,app}$ and M_w/M_n were determined by DMF GPC.

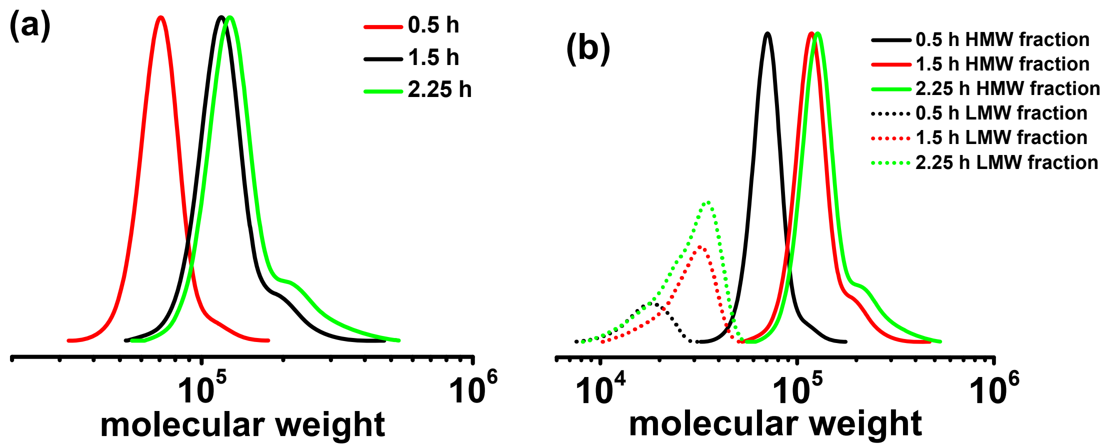

Figure S3. GPC traces of Trox-(P*t*BA-Br)$_{10}$ polymer samples withdrawn periodically during polymerization with DP_{target} = 200: (**a**) high-molecular weight (HMW) fraction of Trox-(P*t*BA-Br)$_{10}$ and (**b**) with an additional low-molecular weight (LMW) fraction formed during the polymerization.

Table S5. Low-molecular weight (LMW) fraction in the samples withdrawn periodically during polymerization of *t*BA from Trox-Br_{10} with DP_{target} = 100.

Sample	% content	$M_{n,app}$[1] (x10^{-3})	M_w/M_n[1]
1	11.23	16.9	1.058
2	22.95	27.3	1.078
3	32.09	27.0	1.116

[1]Apparent $M_{n,app}$ and M_w/M_n were determined by DMF GPC.

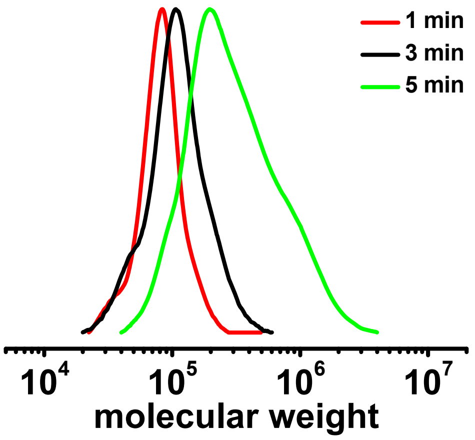

Figure S4. GPC traces of Trox-$(PDMAEMA\text{-}Br)_{10}$ kinetics polymer samples.

Table S6. Kinetics polymer samples of DMAEMA polymerization from Trox-Br_{10}.

Sample	Polymerization time [h]	Conv [%]	$DP_{n,theo}$[1] (per chain)	$M_{n,app}$[2] ($x10^{-3}$)	M_w/M_n[2]	d_{number}[3] [nm]
1	0.017	6	49	76.8	1.15	2.9±0.2
2	0.050	9	73	95.7	1.26	3.3±0.5
3	0.083	12	98	201.9	1.71	7.2±0.9

[1]Monomer conversion (Conv) and theoretical degree of polymerization ($DP_{n,theo}$) calculated according to ^{1}H NMR analysis; [2]Apparent $M_{n,app}$ and M_w/M_n were determined by DMF GPC; [3]Number mean diameter (d_{number}) of PDMAEMA-based polymers measured by DLS (in deionized water) after purification (Figure S10).

S4. Proton nuclear magnetic resonance (^{1}H NMR) spectroscopy analysis of P*t*BA-, PAA- and PDMAEMA-based macromolecules

The structures of the prepared macromolecules with troxerutin core and P*t*BA-, PAA- and PDMAEMA side chains were confirmed by ^{1}H NMR analysis (Figure S5-S7, respectively). Figure S5 showed ^{1}H NMR spectrum for troxerutin-based polymers with P*t*BA side chains (Table 1, entry 3). The chemical shifts for *t*BA units were assigned: δ (ppm) = 1.27–1.94 (11H, CH_3–, –CH_2–, a + β, respectively) and 2.09–2.45 (1H, –CH–, α) [2]. The identified chemical shifts attributed to the characteristic groups of the *t*BA units indicate the presence of polymer chains.

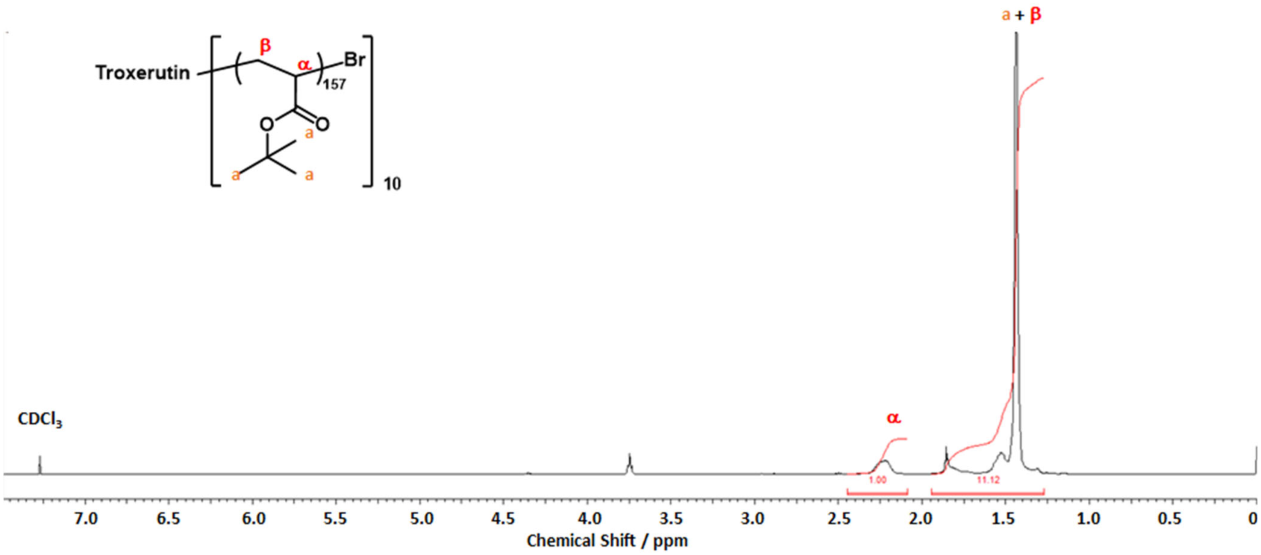

Figure S5. ^{1}H NMR spectrum of macromolecules with troxerutin core and P*t*BA side chains (Table 1, entry 3; M_n = 203,300, M_w/M_n = 1.09) after purification (in $CDCl_3$).

After modification of *t*BA moieties by acidolysis to receive AA groups, the partial disappearance of the characteristic strong peak for methyl groups protons of *t*BA units at ~1.44 ppm is visible (Figure S6). Therefore, the chemical shift at ~12.00 ppm attributed to hydroxyl groups of prepared AA moieties occurs [2], proving the successfully conducted hydrolysis of P*t*BA polymer chains.

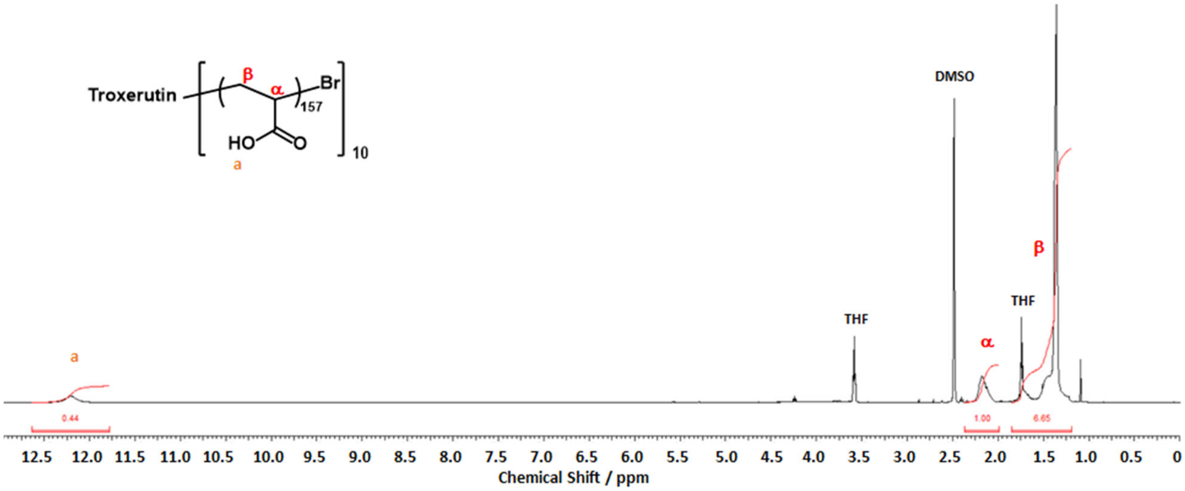

Figure S6. ^{1}H NMR spectrum of macromolecules with troxerutin core and PAA side chains received as a result of acidolysis of *t*BA moieties (Table 1, entry 3; M_n = 203,300, M_w/M_n = 1.09) after purification (in $CDCl_3$).

The ^{1}H NMR spectrum of PDMAEMA-based polymers shows the chemical shifts, 0.66–1.17, 1.69–2.08, 2.20–2.46, 2.54–2.79, and 3.97–4.24 ppm, that are mainly attributable to the $–CH_3$ (3H, α), $–CH_2–$ (2H, β), $–CH_3$ (6H, c), $–CH_2–$ (2H, b), and $–OCH_2–$ (2H, a) groups of the PDMAEMA units in the side chains, respectively [1].

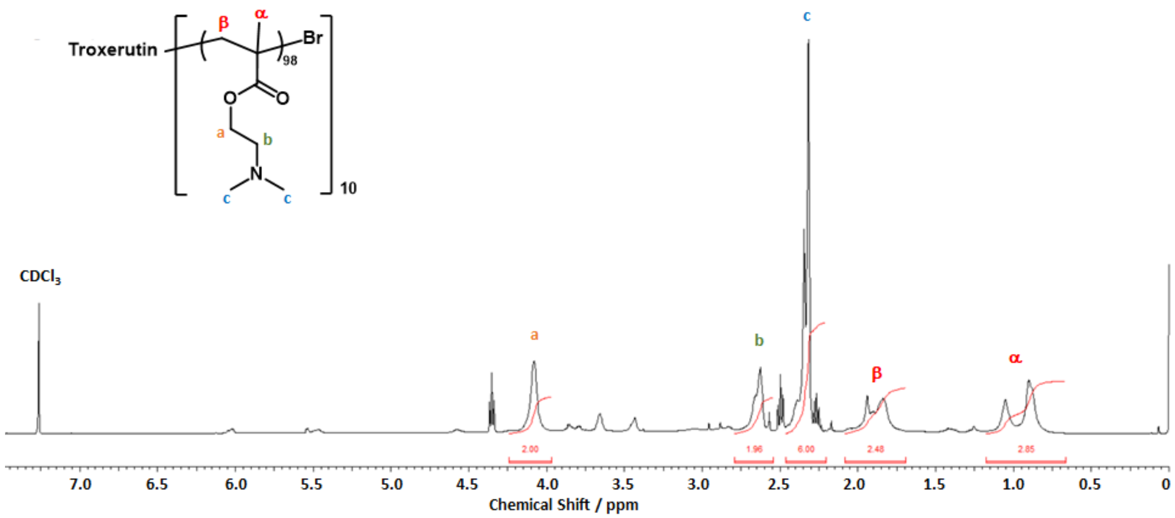

Figure S7. ^{1}H NMR spectrum of macromolecules with troxerutin core and PDMAEMA side chains (Table 1, entry 3; M_n = 203,300, M_w/M_n = 1.09) after purification (in $CDCl_3$).

S5. Fourier-transform infrared spectroscopy (FT-IR) analysis of star-like polymers

FT-IR spectroscopy was used to investigate the chemical modification troxerutin with P*t*BA, PAA and PDMAEMA polymer chains (Figure S8). Brominated troxerutin is characterized by signal within 1,700–1,800 cm^{-1} and 1,000–1,150 cm^{-1}, which are associated with C=O and C–O–C stretching vibrations in ester groups of incorporated bromine structure, respectively. Additionally, the bands at about 1,220–1,350 cm^{-1} and 2,950–3,050 cm^{-1} region related to C–H bending and stretching vibrations $-CH_3$ groups of brominated molecules were noticed [4].

The modified troxerutin core with P*t*BA chains are characterized by a significant band in the 2,950–3,050 cm^{-1} region from stretching C–H vibrations from the ubiquitous $-CH_3$ groups in polyacrylates. Stretching C=O and C–O–C vibrations in ester groups give the bands in 1,700–1,800 cm^{-1} and 1,050–1,200 cm^{-1}, respectively. Typical absorption bands for P*t*BA is also located within 1,380–1,450 cm^{-1} region, corresponding to C–H bending vibrations from $-CH_3$ groups of *t*BA. After the transformation of *t*BA moieties into AA groups, significant differences in the 3,000–3,700 cm^{-1} region is visible. A strong absorption between 2,500 and 3,600 cm^{-1} of carboxyl groups appeared. Additionally, the bands located in 1,050–1,200 cm^{-1} characteristic for stretching C–O–C vibrations in ester groups diapered [2].

Trox-(PDMAEMA-Br)$_{10}$ gives characteristic bands at 1,750 cm^{-1} that can be assigned to C=O stretching vibration. Moreover, C−H(−N(CH_3)$_2$) stretching vibrations gives the band between 2,770–2,940 cm^{-1} as well as the N(CH_3)$_2$ deformational stretching vibrations around 1,459 cm^{-1}. The characteristic bands of the copolymer can be noted to the deformational stretching vibrations of the secondary amine functional group at 2,750–2,800 cm^{-1}, and the stretching vibration of C−N bond around 1,120–1,200 cm^{-1}, related to the DMAEMA content in the structure [5].

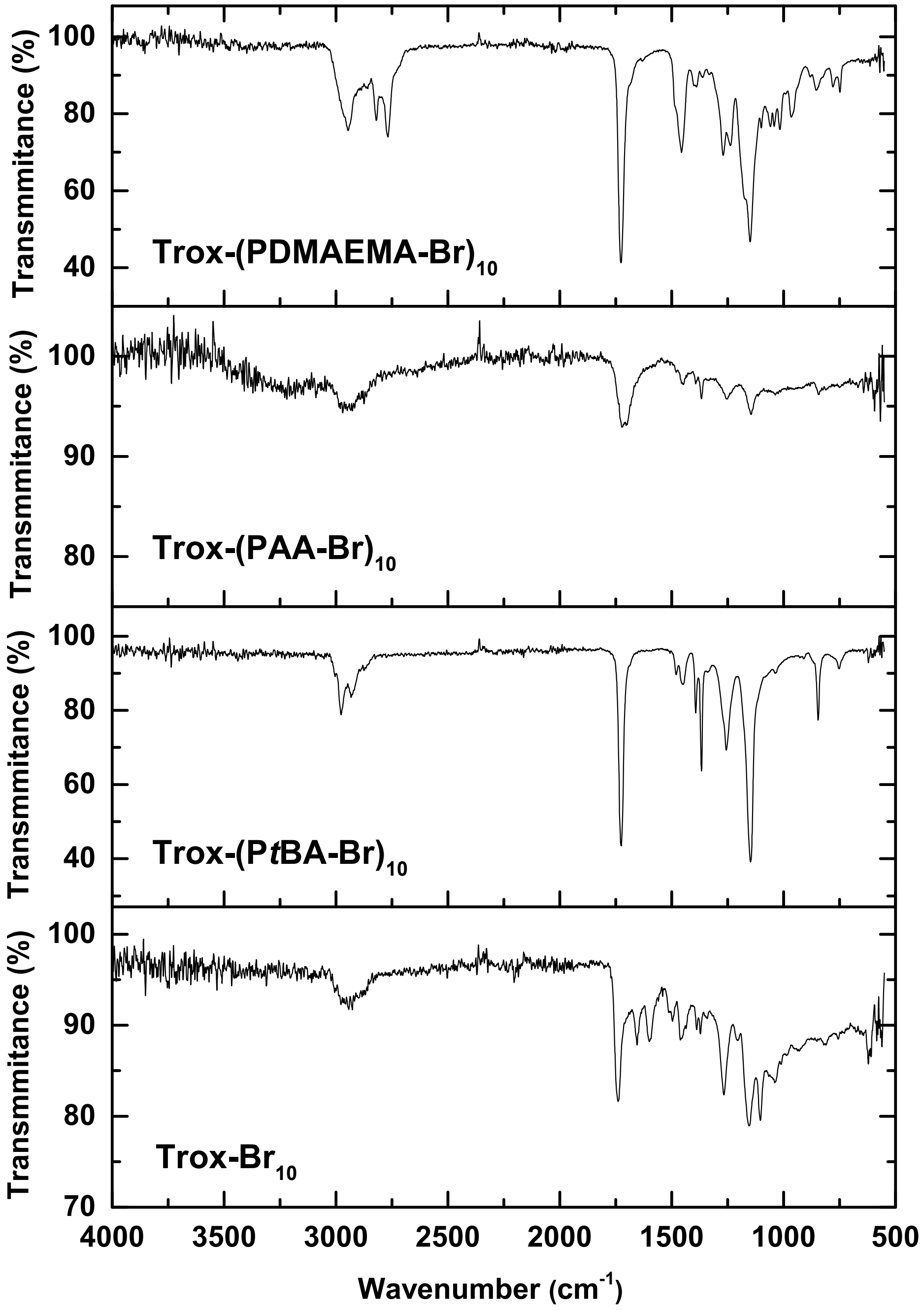

Figure S8. FT-IR spectra of troxerutin modified with bromine (ATRP macroinitiator), P*t*BA (Table 1, entry 3), PAA (hydrolyzed, Table 1, entry 3) and PDMAEMA (Table 1, entry 4).

S6. Hydrodynamic diameter measurements of troxerutin-based polymers by dynamic light scattering (DLS) analysis

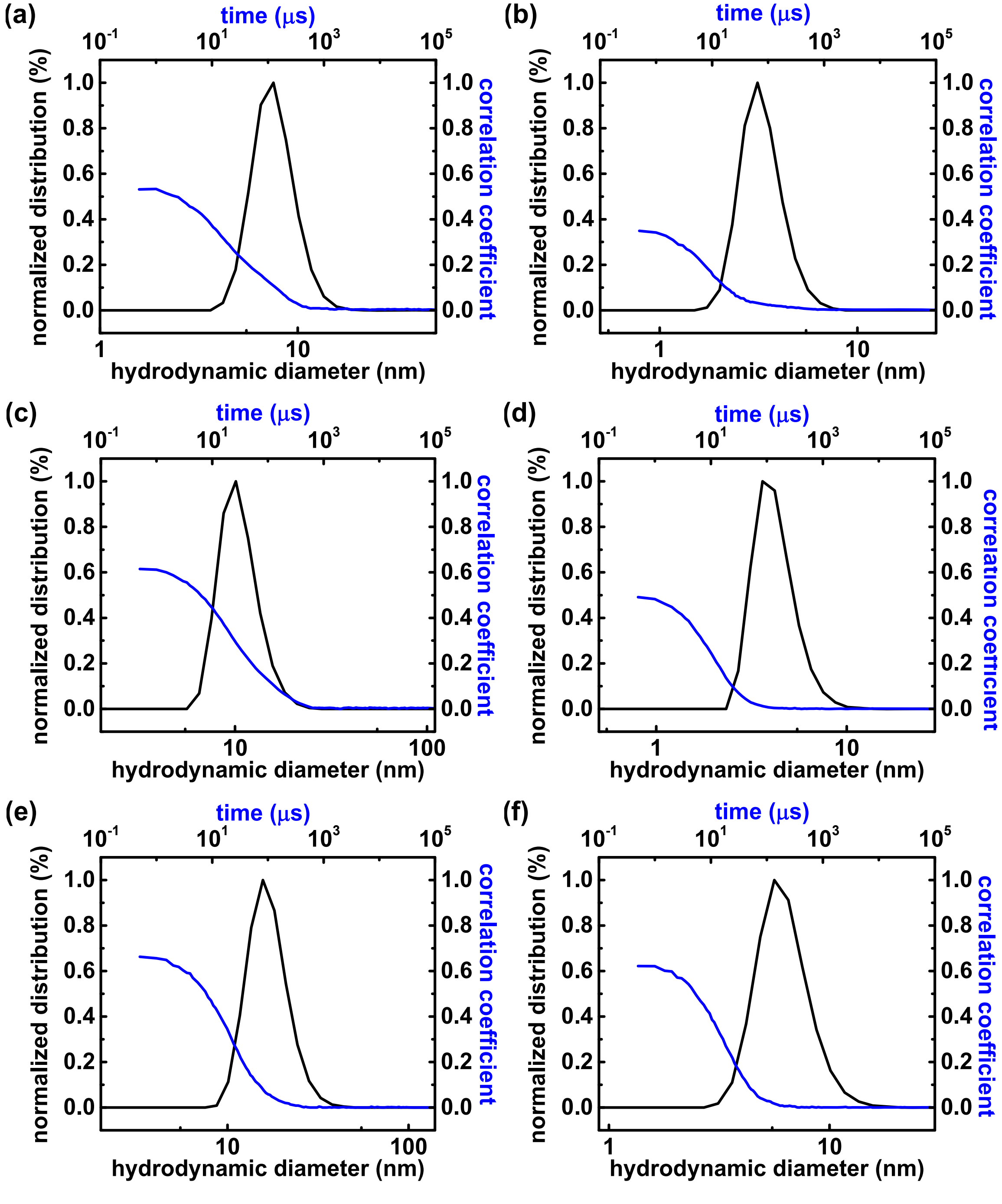

Figure S9. DLS analysis of P*t*BA (a), (c), (e) and corresponding PAA (b), (d), (f) received in the polymerization with the DP_{target} = 50 (Table 1, entry 1), 100 (Table 1, entry 2) and 200 (Table 1, entry 3), respectively.

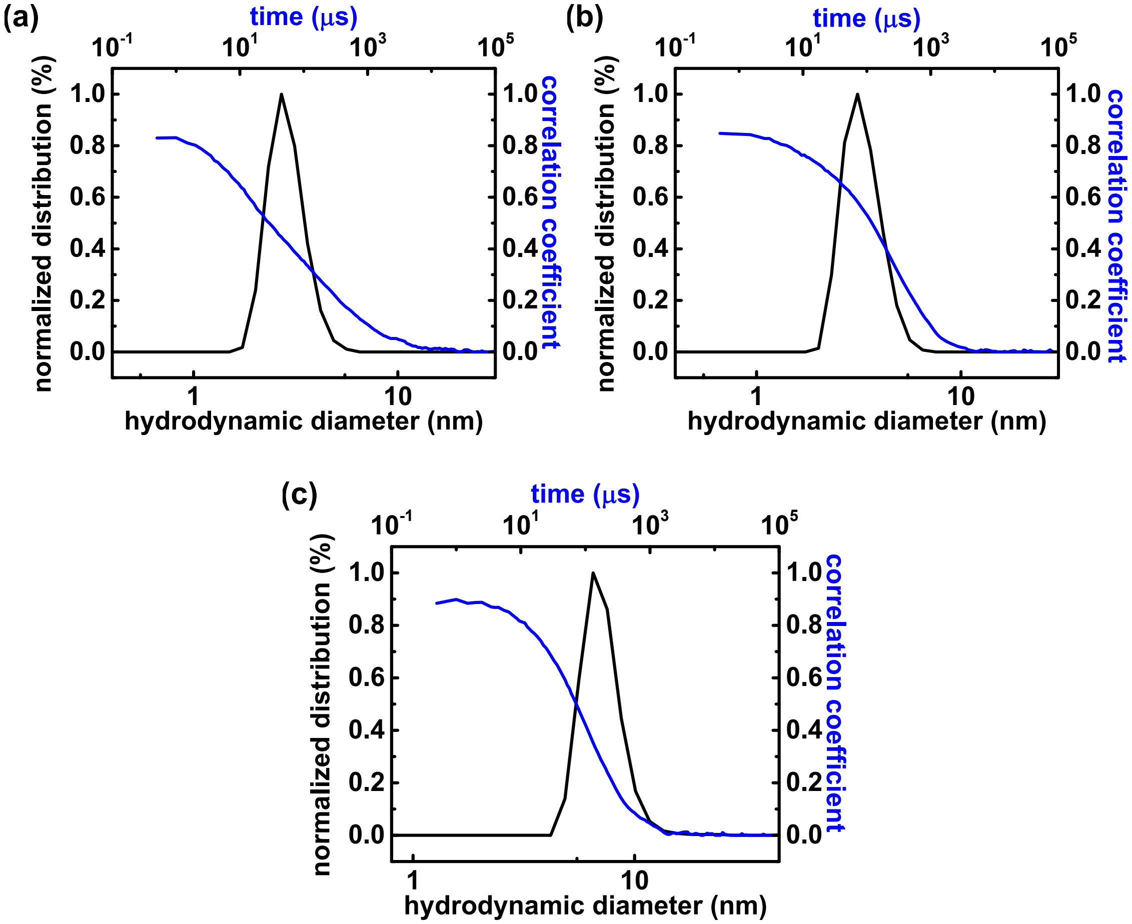

Figure S10. DLS analysis of PDMAEMA-based macromolecules with side chains composed of (a) 49 (Table S6, entry 1), (b) 73 (Table S6, entry 2) and (c) 98 (Table 1, entry 4, Table S6, entry 3) DMAEMA subunits.

S7. Stimuli-responsive behavior of troxerutin-based polymers

Table S7. Number mean diameter of PAA-based macromolecules at varying pH[a).]

Sample	pH	Hydrodynamic diameter [nm]
1	13	13.73 ± 1.52
2	11	13.16 ± 1.31
3	10	11.56 ± 1.31
4	9	11.9 ± 2.06
5	8	12.78 ± 1.5
6	7	11.65 ± 1.68
7	6	13.16 ± 0.71
8	5	648.5 ± 30.35
9	4	1,008 ± 95.1
10	3	3,081 ± 113.6
11	2	5,362 ± 250.9

[a)]The experiment was conducted for the polymer sample received according to Table 1, entry 3 after acidic hydrolysis.

Table S8. Number mean diameter of PDMAEMA-based macromolecules at varying pH[a).]

Sample	pH	Hydrodynamic diameter [nm]
1	13	9.77 ± 0.30
2	11	9.34 ± 0.50
3	10	9.81 ± 0.86
4	9	9.05 ± 0.72
5	8	9.90 ± 0.56
6	7	8.09 ± 1.61
7	6	9.72 ± 0.84
8	5	10.66 ± 1.08
9	4	12.25 ± 1.00
10	3	13.96 ± 1.62
11	2	15.06 ± 1.64

[a)]The experiment was conducted for the polymer sample received according to Table 1, entry 4.

S8. Preparation of PAA- and PDMAEMA-based micelles

Table S9. Loading efficiency (E_L) values of QC in PAA- and PDMAEMA-based micelles.

Sample		Polymer	EL (wt%)
According to Table 1	1	Trox-(PAA-Br)$_{10}$	85
	2		93
	3		86
According to Table S6	1	Trox-(PDMAEMA-Br)$_{10}$	69
	2		74
	3		30

References

1. Chmielarz, P.; Krys, P.; Wang, Z.; Wang, Y.; Matyjaszewski, K. Synthesis of well-defined polymer brushes from silicon wafers *via* surface-initiated *se*ATRP. *Macromol. Chem. Phys.* **2017**, *218*, 1700106, doi:10.1002/macp.201700106.
2. Zaborniak, I.; Macior, A.; Chmielarz, P. Stimuli-responsive rifampicin-based macromolecules. *Materials* **2020**, *13*, 3843, doi:10.3390/ma13173843.
3. Chmielarz, P.; Fantin, M.; Park, S.; Isse, A.A.; Gennaro, A.; Magenau, A.J.D.; Sobkowiak, A.; Matyjaszewski, K. Electrochemically mediated atom transfer radical polymerization (*e*ATRP). *Prog. Polym. Sci.* **2017**, *69*, 47–78, doi:10.1016/j.progpolymsci.2017.02.005.
4. Zaborniak, I.; Chmielarz, P. Miniemulsion switchable electrolysis under constant current conditions. *Polym. Adv. Technol.* **2020**, *31*, 2806–2815, doi:10.1002/pat.5007.
5. Hu, Y.; Wang, J.; Zhang, H.; Jiang, G.; Kan, C. Synthesis and characterization of monodispersed P(St-*co*-DMAEMA) nanoparticles as pH-sensitive drug delivery system. *Mater. Sci. Eng. C* **2014**, *45*, 1–7, doi:10.1016/j.msec.2014.08.061.
